# Supplementary material for: Dynamic Macronutrient Meal-Equivalent Menu Method: Towards Individual Nutrition Intervention Programs
Source: Methods Protoc. 2019 Sep 5;2(3):78. doi: 10.3390/mps2030078 (PMC6789454; doi:10.3390/mps2030078)
Supplement: Supplementary file 1 [file mps-02-00078-s001.zip › Material Suplementario/Table S2.pdf]

Schedule Food Group	No. SFS/d	8:00 a.m. Breakfast No. SFS/d	11:00 p.m. Snack 1 No. SFS/d	2:00 p.m. Lunch No. SFS/d	5:00 p.m. Snack 2 No. SFS/d	8:00 p.m. Dinner No. SFS/d
1. Vegetables	3	0	0	2	0	1
2. Fruits	6	2	1	2	1	0
3. Cereals						
A) Non-fat	3	0	0	2	0	1
B) With fat	2	2	0	0	0	0
4. Legumes	1	0	0	1	0	0
5. Animal Origin (AO)						
A) Very low fat	1	0	0	0	0	1
B) Low fat	2	0	0	2	0	0
C) Moderate fat	0	0	0	0	0	0
D) High fat	0	0	0	0	0	0
6. Milk						
A) Low fat	0	0	0	0	0	0
B) Reduced fat	0	0	0	0	0	0
C) Whole	0	0	0	0	0	0
D) With sugar	0	0	0	0	0	0
7. Fats						
A) Without protein	4	1	0	2	0	1
B) With protein	2	0	1	0	1	0
8. Sugars						
A) Non-fat	3.5	1.5	1	1	0	0
B) With fat	0	0	0	0	0	0

i.e. same individual example as in Table 1. Standard food servings (SFS) according to the Mexican food equivalent exchange lists (SMAE) [27].
