# Supplementary material for: Dynamic Macronutrient Meal-Equivalent Menu Method: Towards Individual Nutrition Intervention Programs
Source: Methods Protoc. 2019 Sep 5;2(3):78. doi: 10.3390/mps2030078 (PMC6789454; doi:10.3390/mps2030078)
Supplement: Supplementary file 1 [file mps-02-00078-s001.zip › Material Suplementario/Table S1.pdf]

Food Group	No. SFS/d	Protein per ^{a}SFS (g)	^{b}Protein in ^{c}ME (g/d)	Fat per ^{a}SFS (g)	^{b}Fat in ^{c}ME (g/d)	Carbohydrates per ^{a}SFS (g)	^{b}Carbohydrates in ^{c}ME (g/d)	Energy per ^{a}SFS (kcal)	^{b}Energy in ^{c}ME (kcal/d)
1. Vegetables	3	2	6	0	0	4	12	25	75
2. Fruits	6	0	0	0	0	15	90	60	360
3. Cereals									
A) Non-fat	3	2	6	0	0	15	45	70	210
B) With fat	2	2	4	5	10	15	30	115	230
4. Legumes	1	8	8	1	1	20	20	120	120
5. Animal Origin (AO)									
A) Very low fat	1	7	7	1	1	0	0	40	40
B) Low fat	2	7	14	3	6	0	0	55	110
C) Moderate fat	0	7	0	5	0	0	0	75	0
D) High fat	0	7	0	8	0	0	0	100	0
6. Milk									
A) Low fat	0	9	0	2	0	12	0	95	0
B) Reduced fat	0	9	0	4	0	12	0	110	0
C) Whole	0	9	0	8	0	12	0	150	0
D) With sugar	0	8	0	5	0	30	0	200	0
7. Fats									
A) Without protein	4	0	0	5	20	0	0	45	180
B) With protein	2	3	6	5	10	3	6	70	140
8. Sugars									
A) Non-fat	3.5	0	0	0	0	10	35	40	140
B) With fat	0	0	0	5	0	10	0	85	0
**TOTAL**	^{d}N/A	^{d}N/A	^{e}51	^{d}N/A	^{f}48	^{d}N/A	^{g}238	^{d}N/A	^{h}1605

i.e. 1600 kcal diet plan, 50g/d protein, 49g/d fat, 240g/d carbohydrates; ^{a}Standard Food Servings (SFS) according to the Mexican food equivalent lists (SAME) [27] which corresponds to the kcal and grams provided by each SFS food group; ^{b}Obtained macronutrient g/d or kcal/d by multiplying SFS content with No. SFS; ^{c}ME: macronutrient meal-equivalent menu; ^{d}N/A: not applicable; Acceptable variations for calculated energy and macronutrient content are: ^{e}protein ±1g/d; ^{f}total fat ±1g/d; ^{g}carbohydrates ±2g/d; ^{h}energy ±15 kcal/d.
