# Supplementary material for: Dynamic Macronutrient Meal-Equivalent Menu Method: Towards Individual Nutrition Intervention Programs
Source: Methods Protoc. 2019 Sep 5;2(3):78. doi: 10.3390/mps2030078 (PMC6789454; doi:10.3390/mps2030078)
Supplement: Supplementary file 1 [file mps-02-00078-s001.zip › Material Suplementario/Figure S1.pdf]

Instructions: each row specifies what to eat at each meal time. You may choose a single box in each row and all within the same meal time are equivalent and exchangeable.

TIME	1	2	3	4	5	6	7
8:00 a.m.	**Banana**<br>- 1 piece (2 Fruit SFS)	**Strawberry Papaya smoothie**<br>- 1 cup strawberries (1 Fruit SFS)<br>- 1 cup papaya (1 Fruit SFS)	**Pear**<br>- 1 piece (2 Fruit SFS)	**Grapes**<br>- 36 pieces (2 Fruit SFS)	**Apple**<br>- 1 piece (1 Fruit SFS)<br>**Kiwi**<br>- 1 piece (1 Fruit SFS)	**Grapefruit**<br>- 1 piece (1 Fruit SFS)<br>**Watermelon**<br>- 1 cup watermelon (1 Fruit SFS)	**Berries**<br>- 1 cup berry mix (1 Fruit SFS)<br>**Melon**<br>- 1 cup melon (1 Fruit SFS)
	**Pancakes**<br>- 2 small pieces of 40g each (2 Cereal B SFS)<br>- 1 tsp. butter (1 Fat A SFS)<br>- 3 tsp. maple syrup (1.5 Sugar A SFS)	**Toasted bread with jam and butter**<br>- 2 whole wheat bread slices<br>- 3 tsp. butter (2 Cereal B SFS + 1 Fat A SFS)<br>- 3 tsp. jam (1.5 Sugar SFS)	**Waffle**<br>- 2 small pieces of 40g each (2 Cereal B SFS)<br>- 1 tsp. butter (1 Fat A SFS)<br>- 3 tsp. maple syrup (1.5 Sugar SFS)	**Toasted bread with jam and cream cheese**<br>- 2 whole wheat bread slices<br>- 3 tbsp. cream cheese (2 Cereal B SFS + 1 Fat A SFS)<br>- 4 tsp. jam (1.5 Sugar SFS)	**Croissant**<br>- 1 piece of 60g (2 Cereal B SFS + 1 Fat A SFS)<br>- 4 tsp. jam (1.5 Sugar SFS)	**Apple Strudel**<br>- 1 piece of 70g (2 Cereal B SFS + 1 Fat A SFS)<br>- 4 tsp. jam (1.5 Sugar SFS)	**Bagel with jam and cream cheese**<br>- 1 whole wheat bagel of 50g<br>- 3 tbsp. cream cheese (2 Cereal B SFS + 1 Fat A SFS)<br>- 4 tsp. jam (1.5 Sugar SFS)
11:00 a.m.	**Grapes**<br>- 18 pieces (1 Fruit SFS)	**Apple**<br>- 1 piece (1 Fruit SFS)	**Melon**<br>- 1 cup melon (1 Fruit SFS)	**Orange**<br>- 2 pieces (1 Fruit SFS)	**Watermelon**<br>- 1 cup watermelon (1 Fruit SFS)	**Tangerine**<br>- 2 pieces (1 Fruit SFS)	**Strawberries**<br>- 1 cup (1 Fruit SFS)
	**Almonds** – 10 pieces<br>(1 Fat B SFS)<br>**Crystallized Fruit** – 3 pieces (1 Sugar SFS)	**Peanut Butter** – 1 tbsp.<br>(1 Fat B SFS)<br>**Marshmallows** – 2 big pieces (15g) (1 Sugar SFS)	**Pistachio** – 18 pieces<br>(1 Fat B SFS)<br>**Lolly Pop** – 1 piece<br>(1 Sugar SFS)	**Honey Roasted Peanuts** – 15 pieces<br>(1 Fat B SFS + 1 Sugar SFS)	**Nuts** – 3 pieces (1 Fat B SFS)<br>**Jelly** – ½ cup (1 Sugar SFS)	**Almond Butter** – 1 tbsp.<br>(1 Fat B SFS)<br>**Mini Marshmallows** – 18 pieces (15g) (1 Sugar SFS)	**Hazelnut Cream** – 1 tbsp.<br>(1 Fat B SFS + 1 Sugar SFS)
2:00 p.m.	**Watermelon Water**<br>- 1 cup watermelon (1 Fruit SFS)<br>- 2 tsp. sugar (1 Sugar SFS)<br>- Free amount of water	**Pineapple-Cucumber Water**<br>- 1 cup pineapple (1 Fruit SFS)<br>- 2 tsp. sugar (1 Sugar SFS)<br>- Free amount of water<br>- ½ cucumber piece (1/2 Vegetable SFS)<br>- ½ lemon piece	**Strawberry Lemonade**<br>- 1 cup strawberries (1 Fruit SFS)<br>- 2 tsp. sugar (1 Sugar SFS)<br>- Free amount of water<br>- 1 lemon piece	**Kiwi Lemonade**<br>- 1 kiwi (1 Fruit SFS)<br>- 2 tsp. sugar (1 Sugar SFS)<br>- Free amount of water<br>- 1 lemon piece	**Tangerine Water**<br>- 2 tangerine pieces (1 Fruit SFS)<br>- 2 tsp. sugar (1 Sugar SFS)<br>- Free amount of water	**Berry Lemonade**<br>- 1 cup berries (1 Fruit SFS)<br>- 2 tsp. sugar (1 Sugar SFS)<br>- Free amount of water<br>- 1 lemon piece	**Orange Water**<br>- 1 orange piece (1 Fruit SFS)<br>- 2 tsp. sugar (1 Sugar SFS)<br>- Free amount of water
	**Avocado Green Salad**<br>- 1/3 avocado piece (1 Fat A SFS)<br>- 1 cup vegetables: spinach, cucumber, bell pepper and alfalfa (1 Vegetable SFS)<br>**Chicken Fajitas**<br>- 50g chicken breast (2 AO A SFS)<br>- 1 tsp. oil (1 Fat A SFS)<br>- 1 cup vegetables: Bell pepper, onion and mushrooms (1 Vegetable SFS)<br>**Whole Beans**<br>- ½ cup (1 Leguminous SFS)<br>**Steamed Pasta**<br>- 1 cup (2 Cereal A SFS)	**Asparagus with Butter**<br>- 1 tsp. butter (1 Fat A SFS)<br>- 1 cup asparagus (1 Vegetable SFS)<br>**Pan Seared Salmon**<br>- 60g salmon (2 AO A SFS)<br>- 1 tsp. oil (1 Fat A SFS)<br>- 1 cup vegetables: spinach and mushrooms (1 Vegetable SFS)<br>**Lentils**<br>- ½ cup (1 Leguminous SFS)<br>**Steamed Pasta**<br>- 1 cup (2 Cereal A SFS)	**Caesar's Salad with Chicken**<br>- 40g chicken breast (1 AO A SFS)<br>- 3 cups lettuce (1 Vegetable SFS)<br>- 2 tbsp. parmesan cheese (1 AO A SFS + ½ Fat SFS)<br>- 1 tbsp. ranch dressing (2 Fat SFS)<br>- 1 cup bread croutons (2 Cereal SFS)<br>**Garlic-cilantro black bean salad**<br>- Free amount garlic and cilantro<br>- ½ cup beans (1 Leguminous SFS)	**Chickpea Salad**<br>- 1 cup vegetables: carrot, mushrooms, tomato, (1 Vegetable SFS)<br>- ½ cup chickpea (1 Leguminous SFS)<br>- 1 tsp. olive oil (1 Fat A SFS)<br>- 1/3 avocado piece (1 Fat A SFS)<br>**Lasagna**<br>- 1 cup of steamed pasta (2 Cereal SFS)<br>- 30g cooked ground beef (1 AO A SFS)<br>- 40g mozzarella (1 AO A SFS)<br>- ½ cup tomato sauce (1 Vegetable SFS)	**Spinach with whole beans**<br>- ½ cup beans (1 Leguminous SFS)<br>- 2 tsp. oil (2 Fat A SFS)<br>- 1 cup vegetables: spinach, onion and garlic (1 Vegetable SFS)<br>**Chicken noodle soup**<br>- 50g chicken breast (2 AO A SFS)<br>- 1 cup vegetables: carrot, pumpkin, Garlic, onion and celery (1 Vegetable SFS)<br>- 1 cup noodles (2 Cereal A SFS)	**Lentils**<br>- ½ cup (1 Leguminous SFS)<br>**Chicken Stew**<br>- 50g chicken breast (2 AO A SFS)<br>- 2 tsp. avocado oil (2 Fat A SFS)<br>- 1 cup steamed vegetables (carrot, onion, pea, tomato) (2 Vegetable SFS)<br>- 1 baked potato (2 Cereal A SFS)	**Avocado Salad**<br>- 1/3 avocado piece (1 Fat A SFS)<br>- 2 cups vegetables: spinach, cucumber, tomato, mushrooms, bell pepper and alfalfa (2 Vegetable SFS)<br>**Lemon pepper fish**<br>- 80g tilapia (2 AO A SFS)<br>- Free amount lemon juice, garlic and pepper<br>- 1 tsp. olive oil (1 Fat A SFS)<br>**Cooked brown rice**<br>- 2/3 cup (2 Cereal A SFS)<br>**Whole Beans**<br>- ½ cup (1 Leguminous SFS)
	**Apple**<br>- 1 piece (1 Fruit SFS)	**Melon**<br>- 1 cup melon (1 Fruit SFS)	**Orange**<br>- 2 pieces (1 Fruit SFS)	**Watermelon**<br>- 1 cup watermelon (1 Fruit SFS)	**Tangerine**<br>- 2 pieces (1 Fruit SFS)	**Strawberries**<br>- 1 cup (1 Fruit SFS)	**Grapes**<br>- 18 pieces (1 Fruit SFS)
5:00 p.m.	**Orange**<br>- 2 pieces (1 Fruit SFS)	**Watermelon**<br>- 1 cup watermelon (1 Fruit SFS)	**Tangerine**<br>- 2 pieces (1 Fruit SFS)	**Strawberries**<br>- 1 cup (1 Fruit SFS)	**Grapes**<br>- 18 pieces (1 Fruit SFS)	**Apple**<br>- 1 piece (1 Fruit SFS)	**Melon**<br>- 1 cup melon (1 Fruit SFS)
	**Pistachios** – 18 pieces (1 Fat B SFS)	**Nuts** – 3 pieces (1 Fat B SFS)	**Peanuts – 15 pieces** (1 Fat B SFS)	**Almond Butter** – 1 tbsp.<br>(1 Fat B SFS)	**Almonds**– 10 pieces<br>(1 Fat B SFS)	**Peanut Butter** – 1 tbsp.<br>(1 Fat B SFS)	**Sesame**– 4 tsp.<br>(1 Fat B SFS)
8:00 p.m.	**Tomato** – 1 piece (1 Vegetable SFS)	**Cucumber** – 1 cup (1 Vegetable SFS)	**Lettuce** – 3 cups (1 Vegetable SFS)	**Spinach** – 1 cup (1 Vegetable SFS)	**Carrot** – 1 cup (1 Vegetable SFS)	**Bell pepper** – 1 cup (1 Vegetable SFS)	**Alfalfa – 1 cup** (1 Vegetable SFS)
	**Boiled egg**<br>- 2 egg whites (1 AO A SFS)<br>**Toasted bread with butter**<br>- 1 slice of whole bread (1 Cereal A SFS)<br>- 1 tbsp. butter (1 Fat A SFS)	**Avocado Tuna**<br>-30g tuna (1 AO A SFS)<br>- 1/3 avocado (1 Fat A SFS)<br>- 1 slice whole bread (1 Cereal A SFS)	**Sandwich**<br>- 25g ham (1 AO A SFS)<br>- 1 tsp. mayonnaise (1 Fat A SFS)<br>- 1 slice whole bread (1 Cereal A SFS)	**Cheese Avocado sandwich**<br>-40g low fat cheese mozzarella (1 AO A SFS)<br>- 1/3 avocado (1 Fat A SFS)<br>- 1 slice whole bread (1 Cereal A SFS)	**Sushi**<br>-30g tuna (1 AO A SFS)<br>- 1/3 avocado (1 Fat A SFS)<br>- 1/3 cup rice (1 Cereal A SFS)	**Flat Bread Pizza**<br>-40g low fat cheese mozzarella (1 AO A SFS)<br>- 1 bacon slice (1 Fat A SFS)<br>- ½ flat bread – (1 Cereal A SFS)	**Wrap Avocado**<br>-40g low fat cheese mozzarella (1 AO A SFS)<br>- 1/3 avocado (1 Fat A SFS)<br>- ½ flat bread (1 Cereal A SFS)
