# Supplementary material for: Dynamic Macronutrient Meal-Equivalent Menu Method: Towards Individual Nutrition Intervention Programs
Source: Methods Protoc. 2019 Sep 5;2(3):78. doi: 10.3390/mps2030078 (PMC6789454; doi:10.3390/mps2030078)
Supplement: Supplementary file 1 [file mps-02-00078-s001.zip › Material Suplementario/Figure S2.pdf]

Patient's ID/Name: ____ Date: _____________________

Basal Energy Expenditure: ______________ kcal/day

Caloric Restriction: ________ kcal/day

Caloric Addition: __________ kcal/day

Total Energy Dietary Plan: ______________ kcal/day

Protein intake: ________ g/kg/day

Theoretical Dietary Resume Table

Macronutrient	%	Grams	Energy (kcal)
1. Protein			
2. Fats			
3. Carbohydrates			
TOTAL	100	-	

Mealtimes, schedule and standard food servings per day (SFS/d) individual distribution. Number of standard food servings per day, energy and macronutrient calculation format in the macronutrient meal-equivalent menu method.

Food Group	No. SFS/d	Protein per [a]SFS (g)	[b]Protein in [c]ME (g/d)	Fat per [a]SFS (g)	[b]Fat in [c]ME (g/d)	Carbohydrates per [a]SFS (g)	[b]Carbohydrates in [c]ME (g/d)	Energy per [a]SFS (kcal)	[b]Energy in [c]ME (kcal/d)
1. Vegetables		2		0		4		25	
2. Fruits		0		0		15		60	
3. Cereals									
A) Non-fat		2		0		15		70	
B) With fat		2		5		15		115	
4. Legumes		8		1		20		120	
5. Animal Origin (AO)									
A) Very low fat		7		1		0		40	
B) Low fat		7		3		0		55	
C) Moderate fat		7		5		0		75	
D) High fat		7		8		0		100	
6. Milk									
A) Low fat		9		2		12		95	
B) Reduced fat		9		4		12		110	
C) Whole		9		8		12		150	
D) With sugar		8		5		30		200	
7. Fats									
A) Without protein		0		5		0		45	
B) With protein		3		5		3		70	
8. Sugars									
A) Non-fat		0		0		10		40	
B) With fat		0		5		10		85	
**TOTAL**	[d]N/A	[d]N/A		[d]N/A		[d]N/A		[d]N/A	

[a]Standard Food Servings (SFS) according to the Mexican food equivalent lists (SMAE) [27] which corresponds to the kcal and grams provided by each SFS food group; [b]Obtained macronutrient g/d or kcal/d by multiplying SFS content with No. SFS; [c]ME: macronutrient meal-equivalent menu; [d]N/A: not applicable; Acceptable variations for calculated energy and macronutrient content are: [e]protein ±1g/d; [f]total fat ±1g/d; [g]carbohydrates ±2g/d; [h]energy ±15 kcal/d.

Mealtimes, schedule and standard food servings per day (SFS/d) individual distribution planification sheet.

Number of meals per day regularly done by the patient: __________ Number of meals suggested per day: __________

Schedule						
Food Group	No. SFS/d	Meal 1 No. SFS/d	Meal 2 No. SFS/d	Meal 3 No. SFS/d	Meal 4 No. SFS/d	Meal 5 No. SFS/d
1. Vegetables						
2. Fruits						
3. Cereals						
A) Non-fat						
B) With fat						
4. Legumes						
5. Animal Origin (AO)						
A) Very low fat						
B) Low fat						
C) Moderate fat						
D) High fat						
6. Milk						
A) Low fat						
B) Reduced fat						
C) Whole						
D) With sugar						
7. Fats						
A) Without protein						
B) With protein						
8. Sugars						
A) Non-fat						
B) With fat						

Patient's ID/Name: ____ **Date:** ____________________

Instructions: each row specifies what to eat at each meal time. You may choose a single box in each row and all within the same meal time are equivalent and exchangeable.

TIME	1	2	3	4	5	6	7
_____							
_____							
_____							
_____							
_____							
_____							
